# Post-radiotherapy xerostomia and quality of life in head and neck cancer patients

**DOI:** 10.1007/s00520-026-10948-9

**Published:** 2026-06-27

**Authors:** Georg Hoene, Lucie Carlotta Waldzus, Boris Schminke, Lennart Johannes Gruber, Leif Dröge, Henning Schliephake, Susanne Wolfer

**Affiliations:** 1 Clinic for Oral and Maxillofacial Surgery, University Medical Center Goettingen, Robert-Koch-Strasse 40, 37075 Goettingen, Germany; 2 Department of Radiotherapy and Radiation Oncology, University Medical Center, Goettingen, Germany

**Keywords:** Head and neck cancer, Radiotherapy, Xerostomia, Salivary gland hypofunction, Oral health-related quality of life (OHRQoL), OHIP-G14

## Abstract

**Purpose:**

Radiotherapy for head and neck cancer frequently results in radiation-induced xerostomia, a chronic symptom that can persist long-term and compromise oral function and survivorship.

**Methods:**

A cross-sectional, non-interventional survey was conducted using a mixed recruitment approach (department-based and nationwide online dissemination). Xerostomia prevalence, symptom characteristics, coping strategies, and oral health-related quality of life (OHRQoL) were assessed using a study-specific xerostomia questionnaire and the validated OHIP-G14 instrument. Overall, 253 questionnaires were returned (202 complete and 51 partial), including 163 completed online via LimeSurvey and 39 completed on paper during routine follow-up visits at the tumour clinic of the department.

**Results:**

Xerostomia was reported by 90% of respondents; 53.1% described the daily burden as strong/very strong. Patients with xerostomia had significantly worse OHRQoL than those without (mean OHIP-G14 26.7 vs. 17.3; *p* < 0.001), corresponding to a clinically relevant mean difference of 9.4 points. In multivariable linear regression, xerostomia remained independently associated with worse OHRQoL (B = 8.86, *p *< 0.001). Functional impairment and oral pain were more pronounced than psychosocial or aesthetic domains, and perceived xerostomia severity correlated with OHIP scores. Commonly used coping strategies included frequent water intake (81%), sugar-free gum/lozenges (46%), saliva substitutes (43%), and home remedies (e.g., oil or tea) (33%); prescription sialogogues were infrequently used (15%) and perceived as less effective.

**Conclusion:**

Post-radiotherapy xerostomia is highly prevalent and independently associated with clinically meaningful OHRQoL impairment, underscoring the need for optimized counselling and structured supportive care. Management was dominated by low-threshold behavioural strategies, while prescription sialogogues were used by only a minority of respondents and were rated less effective in terms of perceived benefit.

**Supplementary information:**

The online version contains supplementary material available at 10.1007/s00520-026-10948-9.

## Introduction

Head and neck cancers comprise a heterogeneous group of malignancies arising from the oral cavity, pharynx, larynx, nasal cavity and paranasal sinuses. Histopathologically, approximately 90% are squamous cell carcinomas, representing one of the most common cancer entities worldwide [1, 2]. Established risk factors include tobacco exposure and alcohol consumption, while the increasing incidence of HPV-associated disease—particularly oropharyngeal carcinoma—has shifted epidemiological patterns in many regions [1, 3, 4].

Radiotherapy is one of the cornerstones of curative treatment concepts, either as an adjuvant modality following surgery or as definitive therapy (often combined with systemic treatment) [5]. Technical advances such as intensity-modulated radiotherapy (IMRT) and volumetric modulated arc therapy (VMAT) have improved target conformity and reduced dose to organs at risk, thereby enhancing functional preservation [6–8]. Nevertheless, oral late toxicities remain clinically relevant and can substantially impair long-term survivorship outcomes [9, 10].

Radiation-induced xerostomia is among the most frequent and burdensome complications following head and neck radiotherapy. It results from structural and functional damage to major salivary glands and may persist for years or permanently [11, 12]. Importantly, xerostomia denotes a subjective sensation of oral dryness and must be distinguished from objectively measurable hyposalivation [13, 14]. Salivary gland hypofunction compromises lubrication, bolus formation and taste perception and predisposes to secondary complications such as mucosal lesions, caries and infections, thereby affecting fundamental daily functions including eating, speaking, sleeping and social interaction [10, 15–17]. Consequently, xerostomia is increasingly conceptualised as a multidimensional symptom complex with substantial implications for oral health-related quality of life (OHRQoL) after completion of oncological therapy [15].

While the pathophysiology and dosimetric determinants of salivary gland injury have been extensively described, fewer data address how patients perceive xerostomia in daily life, which coping strategies they adopt, and how these strategies relate to OHRQoL. Patient-reported outcomes are crucial for capturing the lived burden of late toxicity and for improving needs-based supportive care pathways.

Therefore, the present study aimed to determine the prevalence and perceived severity of post-radiotherapy xerostomia in a heterogeneous cohort of head and neck cancer patients, to quantify its association with OHRQoL using OHIP-G14, and to characterise patient-reported effectiveness of commonly used xerostomia management strategies.

## Materials & methods

### Study design and setting

A cross-sectional, non-interventional survey study assessed patient-reported post-radiotherapy xerostomia, associated symptoms, and oral health-related quality of life (OHRQoL) under routine care conditions, without altering ongoing treatments or assigning participants to intervention groups. Participants were recruited via two complementary pathways: paper questionnaires during routine follow-up visits at the tumour clinic of the Department of Oral and Maxillofacial Surgery, University Medical Center Goettingen, Germany, and nationwide online participation disseminated through head and neck cancer patient advocacy organisations using LimeSurvey.

### Questionnaire development and ethics

A study-specific xerostomia questionnaire was co-developed with the patient advocacy groups Kopf-Hals-M.U.N.D.-Krebs e.V. and the Bundesverband Kehlkopf- und Kopf-Hals-Tumore e.V. to ensure that item wording and response options were understandable, patient-relevant, and feasible to answer in routine survivorship settings. It was submitted together with the study protocol and patient information to the local ethics committee. A positive ethics committee vote was obtained (Ethics Committee of the University Medical Center Goettingen; reference number 26/12/24), after which data collection commenced. The study was prospectively registered in the German Clinical Trials Register (DRKS; DRKS00036144). Participation was voluntary; all participants provided informed consent and could withdraw at any time without consequences.

### Participants: eligibility and recruitment

Eligible participants were adults (≥ 18 years) with a head and neck oncologic diagnosis involving the oral cavity, pharynx, larynx, nose/paranasal sinuses, or salivary glands, who had completed radiotherapy to the head and neck region. Exclusion criteria were ongoing radiotherapy, age < 18 years, or lack of (capacity for) informed consent. Recruitment used a mixed approach to increase heterogeneity: (i) in-person recruitment during routine oncologic follow-up in the tumour clinic of the Department of Oral and Maxillofacial Surgery, University Medical Center Goettingen (with paper questionnaires if preferred) and (ii) nationwide dissemination via the two largest patient support organisations, enabling online participation.

### Data collection procedures and study period

Data were collected between 19 February 2025 and 25 August 2025. The survey was implemented using LimeSurvey® (LimeSurvey GmbH, Hamburg, Germany) and was accessible via desktop and mobile devices. In the department-based pathway, participants completed paper questionnaires during routine follow-up visits at the tumour clinic; these were subsequently entered manually into LimeSurvey to ensure a unified dataset.

Overall, 253 questionnaires were received (202 complete; 51 partially completed). Of these, 214 questionnaires were submitted via LimeSurvey (163 complete; 51 partially completed), and 39 complete paper questionnaires were collected from patients during follow-up visits. Partially completed questionnaires were included if they contained data relevant to the research question; therefore, the number of respondents varied between analyses of individual items. Because the survey was disseminated via multiple recruitment pathways, including nationwide online distribution through patient self-help organisations, a formal overall response rate could not be determined.

### Study-specific xerostomia questionnaire

Xerostomia was assessed using a study-specific structured questionnaire item asking whether participants had experienced dry mouth after radiotherapy (yes/no). Owing to the questionnaire-based design, detailed radiotherapy dose and dosimetric parameters were not collected. Respondents reporting xerostomia completed additional items on symptom onset, perceived everyday burden, time-of-day peak severity, xerostomia-related functional problems, perceived changes in saliva production, sleep-related impact, and xerostomia management strategies. Functional problems and management strategies were assessed using multiple-response items. The full wording of the original German questionnaire is provided in the Supplementary Material (Supplementary Appendix S1), and an English summary of the xerostomia-specific items and response formats is provided in Supplementary Table S1.

### Oral health-related quality of life (OHIP-G14)

OHRQoL was measured using the validated German 14-item short form of the Oral Health Impact Profile (OHIP-G14). The OHIP was originally derived from a 49-item instrument grounded in Locker’s conceptual model [18] and was developed by Slade and Spencer [19]. The German short version comprises 14 items mapping four OHRQoL dimensions (oral function, orofacial pain, orofacial appearance, psychosocial impact). Responses use a five-point Likert frequency scale (0–4), referring to the preceding four weeks, and are summed to a total score ranging from 0 to 56, with higher scores indicating worse OHRQoL.

### Sample size planning

An a priori sample size calculation was conducted using G*Power v3.1.9.2 (University of Duesseldorf, Duesseldorf, Germany) assuming α = 0.05, power = 0.95, and effect size = 0.3, resulting in a minimum required sample size of *n* = 111. To account for incomplete questionnaires and variability, a target of ~ 200 fully analyzable datasets was defined.

### Data management

Responses were exported from LimeSurvey to Microsoft Excel and prepared for statistical analysis in IBM® SPSS® Statistics v31.0 (IBM Corporation, Armonk, NY, USA). Prior to analysis, datasets were checked for completeness, logical consistency, plausibility, and outliers; free-text entries were harmonised into predefined categories where appropriate. Inconsistent responses in the “effectiveness of measures” section were handled by restricting effectiveness analyses to participants who had indicated prior use of the respective measure; inconsistent entries were treated as missing.

### Statistical analysis

Analyses were descriptive and exploratory. Categorical variables were summarized using absolute and relative frequencies; continuous variables were described using mean, standard deviation, median, minimum/maximum, and range. Missing data were handled using available-case analysis for descriptive summaries and complete-case (listwise) analysis for inferential tests and regression models; no imputation was performed. OHIP-G14 total scores were calculated by summation of all 14 items. Distributional assumptions were assessed using Q–Q plots and normality tests (Kolmogorov–Smirnov and Shapiro–Wilk), and homogeneity of variances was evaluated prior to mean comparisons. Depending on scale level and distribution, the following inferential approaches were applied: independent-samples *t*-tests for group mean comparisons, Spearman rank correlations for associations involving non-normally distributed/ordinal variables, Friedman tests for within-subject comparisons across OHRQoL dimensions with Wilcoxon signed-rank post-hoc tests, and multiple linear regression to evaluate xerostomia as a predictor of OHRQoL while adjusting for prespecified covariates (sex, age category, time since radiotherapy, tumour entity, and tumour stage). For multiple pairwise comparisons following Friedman’s test, Bonferroni correction was applied [20]. A two-sided significance level of *p* < 0.05 was used unless otherwise specified by correction procedures. In a post-hoc, exploratory analysis, online- and clinic-recruited participants were compared using Fisher's exact test for categorical variables and the Mann–Whitney U test for continuous variables; given the exploratory nature, no correction for multiple comparisons was applied. Statistical analyses were supported by the Scientific Service Unit “Medical Biometry and Statistical Bioinformatics” (MBSB).

## Results

### Study sample and data completeness – Participant flow

Overall, 253 questionnaires were returned, including 202 complete questionnaires (163 completed online and 39 on paper) and 51 partially completed questionnaires submitted online; responses were included on an item-wise basis depending on data availability.

† Multiple responses permitted; percentages therefore do not sum to 100%

‡ Acute adverse effects were assessed by two binary items (during radiotherapy; after radiotherapy), with optional free-text specification.

### Participant, disease, and treatment characteristics

Baseline characteristics are summarised in Table 1. The small T0 subgroup reflects cases of cancer of unknown primary (CUP), as reported by participants. Most respondents were aged 50–79 years (211/233; 90.6%), with a slightly higher male representation (men 124/233; 53.2%). The most frequently reported tumour entities were oral cavity cancers (*n* = 95; 42%), laryngeal (*n* = 55; 24%) and pharyngeal cancers (*n* = 39; 17%). Most participants completed the survey > 2 years after radiotherapy (143/229; 62.4%). Surgery (192/234; 82.1%) and chemotherapy (126/234; 53.8%) were commonly reported as additional treatments beyond radiotherapy.

**Table 1:** Participant, disease, and treatment characteristics (*N* = 253) Values are n (%) unless stated otherwise. Denominators vary due to item non-response. For multiple-response items, percentages do not sum to 100%

Characteristic	Category	n (%)
**Sociodemographics**		
Age category (years)	*n* = 233	
	< 30	3 (1.3)
	30–39	6 (2.6)
	40–49	7 (3.0)
	50–59	48 (20.6)
	60–69	99 (42.5)
	70–79	64 (27.5)
	≥ 80	6 (2.6)
Sex	*n* = 233	
	Male	124 (53.2)
	Female	108 (46.4)
	Diverse	1 (0.4)
**Disease characteristics**		
Tumour entity/type	*n* = 225	
	Oral cavity cancer	95 (42.2)
	Laryngeal cancer	55 (24.4)
	Pharyngeal cancer	39 (17.3)
	Salivary gland cancer	16 (7.1)
	Lymphoma (neck region)	6 (2.7)
	Nasal cavity/paranasal sinus cancer	4 (1.8)
	Other	10 (4.4)
T stage at diagnosis	*n *= 225	
	T0	5 (2.2)
	T1	32 (14.2)
	T2	26 (11.6)
	T3	70 (31.1)
	T4	54 (24.0)
	Unknown	38 (16.9)
Metastatic status at diagnosis*	*n *= 225	
	Regional nodal metastases reported (N +)	107 (47.6)
	Distant metastases reported (M1)	4 (1.8)
	No metastases reported	106 (47.1)
	Unknown	8 (3.6)
**Treatment characteristics**		
Time since completion of radiotherapy	*n *= 229	
	< 6 months	26 (11.4)
	6–12 months	19 (8.3)
	1–2 years	41 (17.9)
	> 2 years	143 (62.4)
Additional treatments beyond radiotherapy^†^	*n *= 234	
	Surgery	192 (82.1)
	Chemotherapy	126 (53.8)
	Radiotherapy only	10 (4.3)
	Other additional measures	6 (2.6)
**Acute toxicities**^‡^		
Acute adverse effects	*n* = 221	
	During radiotherapy	171 (77.4)
	After radiotherapy	172 (77.8)

* Metastatic status was self-reported and is presented in non-overlapping categories; totals sum to *n* = 225

### Acute toxicities during and after radiotherapy

Acute adverse effects were assessed in *n* = 221 respondents. Acute toxicities were reported by 171/221 (77.4%) during radiotherapy and by 172/221 (77.8%) after radiotherapy. In optional free-text responses, participants most commonly reported pain, dysphagia, loss of taste, nausea/vomiting, and skin or mucosal reactions.

### Xerostomia prevalence, timing, and symptom burden

Radiotherapy-associated xerostomia was assessed in *n* = 220. Xerostomia was reported by 198/220 (90.0%), while 22/220 (10.0%) reported no xerostomia. Among respondents with xerostomia (*n* = 198), onset occurred most frequently during radiotherapy (109/198; 55.1%) or immediately after completion (46/198; 23.2%). Onset was reported weeks after completion by 24/198 (12.1%) and > 6 months after completion by 7/198 (3.5%); 12/198 (6.1%) could not specify onset. Perceived everyday burden of xerostomia was analysed in *n* = 177 valid single-response cases. Due to an unintended multi-select setting for the item assessing everyday burden in the online survey, 21/198 respondents selected more than one burden category; these cases were excluded from this item-specific analysis to preserve interpretability of the ordinal burden scale. Strong burden was reported by 58/177 (32.8%) and very strong burden by 36/177 (20.3%); 62/177 (35.0%) reported moderate burden, 20/177 (11.3%) low burden, and 1/177 (0.6%) no burden.

**Fig. 1:**
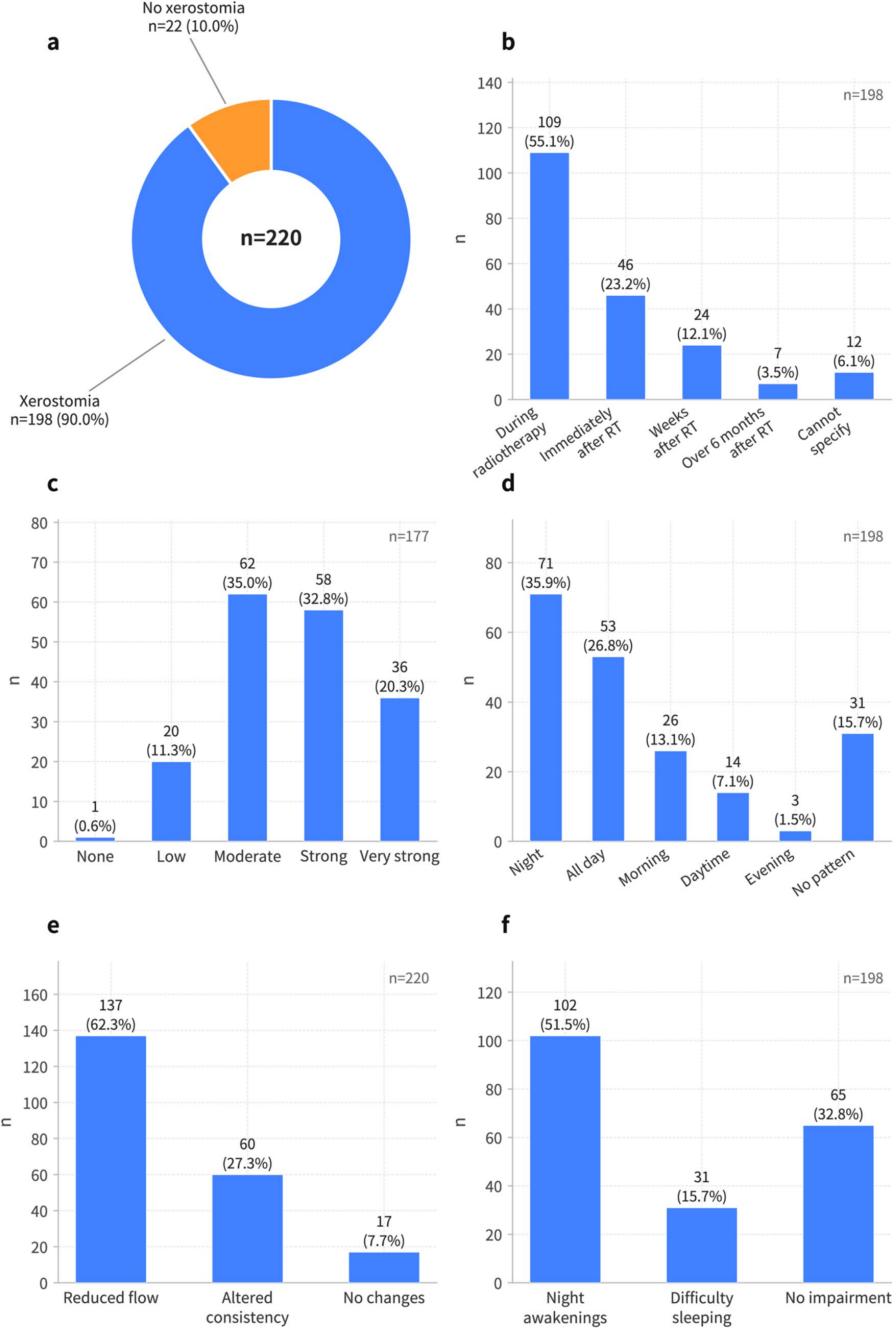
Descriptive characteristics of radiotherapy-associated xerostomia and related symptom profiles. **a** Prevalence of xerostomia among respondents assessed for radiotherapy-associated xerostomia (*n* = 220): xerostomia *n *= 198 (90.0%) and no xerostomia *n *= 22 (10.0%). **b** Timing of xerostomia onset among xerostomia-affected respondents (*n *= 198): during radiotherapy *n* = 109 (55.1%), immediately after completion *n* = 46 (23.2%), weeks after completion *n* = 24 (12.1%), over 6 months after completion *n *= 7 (3.5%), cannot specify *n* = 12 (6.1%). **c** Perceived everyday burden of xerostomia in valid single-response cases (n = 177): none *n*= 1 (0.6%), low *n *= 20 (11.3%), moderate *n* = 62 (35.0%), strong *n *= 58 (32.8%), very strong n = 36 (20.3%); due to an unintended multi-select setting, 21/198 respondents selected more than one category and were excluded from this item-specific analysis. **d** Time of day of maximal dryness among xerostomia-affected respondents (*n* = 198): night *n* = 71 (35.9%), all day *n* = 53 (26.8%), morning *n *= 26 (13.1%), daytime *n* = 14 (7.1%), evening *n* = 3 (1.5%), no pattern *n *= 31 (15.7%). **e** Self-reported salivary changes among respondents assessed (*n *= 220): reduced salivary flow *n *= 137 (62.3%), altered saliva consistency *n *= 60 (28.0%), no changes *n *= 17 (7.8%). **f** Sleep-related impact among xerostomia-affected respondents (*n* = 198): night-time awakenings due to dryness *n *= 102 (51.5%), difficulty falling or staying asleep *n *= 31 (15.7%), no sleep impairment *n* = 65 (32.8%)

Regarding the timing of maximal dryness among xerostomia-affected respondents (*n* = 198), 71/198 (35.9%) reported that dryness was most pronounced at night and 53/198 (26.8%) described symptoms as severe throughout the entire day. Morning predominance was reported by 26/198 (13.1%), daytime predominance by 14/198 (7.1%), evening predominance by 3/198 (1.5%), and no specific temporal pattern by 31/198 (15.7%). Salivary changes were assessed in *n *= 220 respondents: 137/220 (62.3%) reported reduced salivary flow and 60/220 (28.0%) reported altered saliva consistency; 17/220 (7.8%) reported no changes. Sleep-related consequences were analysed separately among xerostomia-affected respondents (*n *= 198): 102/198 (51.5%) reported night-time awakenings due to dryness, 31/198 (15.7%) reported difficulty falling or staying asleep, and 65/198 (32.8%) reported no sleep impairment. Descriptive characteristics of radiotherapy-associated xerostomia and related symptom profiles are visualised in Fig. 1.

### Oral health-related quality of life (OHIP-G14)

**Fig. 2:**
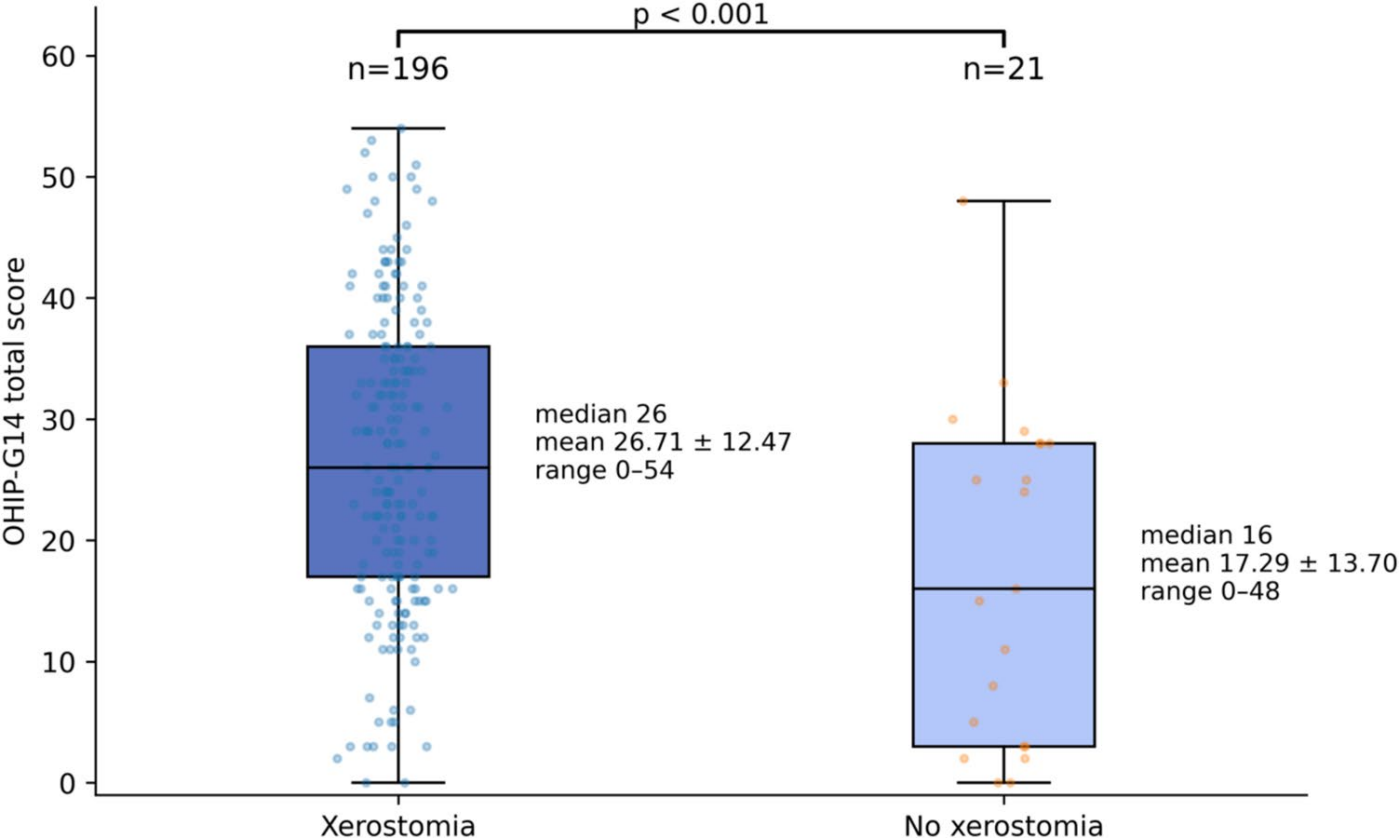
Oral health-related quality of life (OHIP-G14 total score) by xerostomia status (*n* = 217). Participants with xerostomia (*n* = 196) had higher OHIP-G14 scores, indicating worse OHRQoL, than those without xerostomia (*n* = 21). Independent-samples t-test, *p* < 0.001; mean difference 9.42 points (95% CI 3.73–15.13); Levene’s test F = 0.903, *p* = 0.343. Boxplots depict the median and interquartile range; whiskers indicate the observed range (min–max); points represent individual participants

The OHIP-G14 total score differed significantly by xerostomia status (*n* = 217, independent-samples t-test, *p* < 0.001). Participants reporting xerostomia had higher OHIP-G14 scores (mean 26.71, SD 12.47, median 26, range 0–54) than those without xerostomia (mean 17.29, SD 13.70, median 16, range 0–48), corresponding to a mean difference of 9.42 points (95% CI 3.73–15.13). Variance homogeneity was supported by Levene’s test (F = 0.903, *p* = 0.343). (Fig. 2).

In a multivariable linear regression model with the OHIP-G14 sum score as the dependent variable, xerostomia (yes vs. no) remained independently associated with poorer OHRQoL (unstandardized regression coefficient B = 8.86, p = 0.001) after adjustment for sex, age, time since radiotherapy completion, tumour entity, and tumour stage. The model was statistically significant overall (F(22,194) = 4.04, *p* < 0.001) and explained 23.6% of the variance in OHIP-G14 scores (adjusted R^2^ = 0.236). Compared with participants surveyed > 2 years after radiotherapy (reference category), more recent radiotherapy completion was associated with higher OHIP-G14 scores (< 6 months: B = 8.95, *p* = 0.001; 6–12 months: B = 7.17, *p* = 0.017; 12–24 months: B = 5.41, *p* = 0.014), indicating a gradual improvement in OHRQoL over time after treatment.

**Fig. 3:**
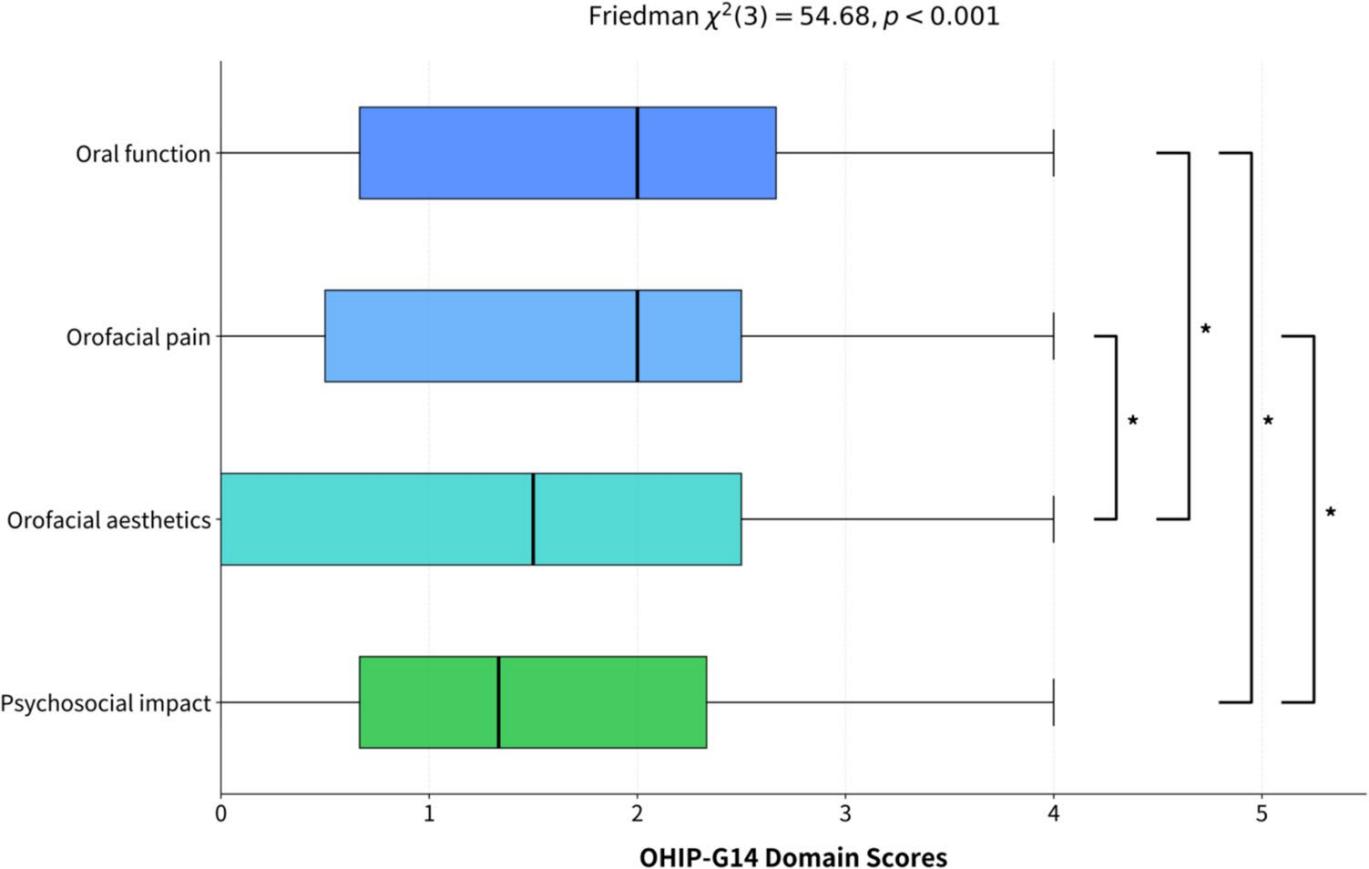
Domain-level OHIP-G14 score distributions among xerostomia-affected respondents (*n* = 196). Horizontal boxplots show the distribution of scores for oral function, orofacial pain, orofacial aesthetics, and psychosocial impact. Overall within-participant differences across domains were significant (Friedman χ^2^(3) = 54.68, *p* < 0.001). Brackets indicate significant pairwise Wilcoxon signed-rank comparisons after Bonferroni adjustment (α = 0.0083). Differences between oral function and orofacial pain and between orofacial aesthetics and psychosocial impact were not significant

Dimension-level analyses among xerostomia-affected respondents showed significant within-participant differences across OHRQoL domains (Friedman χ^2^(3) = 54.68, *p *< 0.001). Oral function and orofacial pain showed higher mean ranks (2.85 and 2.79, respectively) than orofacial aesthetics and psychosocial impact (2.25 and 2.11, respectively). Pairwise Wilcoxon signed-rank tests (Bonferroni-adjusted α = 0.0083) indicated significant differences between oral function and orofacial aesthetics, oral function and psychosocial impact, orofacial pain and orofacial aesthetics, and orofacial pain and psychosocial impact, whereas differences between oral function and orofacial pain and between orofacial aesthetics and psychosocial impact were not significant. The corresponding domain-score distributions and significant pairwise comparisons are shown in Fig. 3.

### Association between perceived xerostomia burden and OHIP-G14

Exploratory Spearman correlation analyses (*n* = 176) showed that greater perceived xerostomia burden was associated with higher OHIP-G14 total scores, indicating that increasing subjective xerostomia burden was linked to poorer oral health-related quality of life. Consistent with this pattern, the categories “strong” (ρ = 0.218, *p* = 0.004) and “very strong” burden (ρ = 0.365, *p* < 0.001) were associated with higher OHIP-G14 scores, whereas “low” (ρ =  − 0.316, *p* < 0.001) and “moderate” burden (ρ =  − 0.322, *p* < 0.001) were associated with lower OHIP-G14 scores.

### Xerostomia management, perceived effectiveness, behavioural strategies, and patient education

Among xerostomia-affected respondents, xerostomia management was analysed in *n* = 189 (multiple responses permitted). Increased water intake was most frequently reported (153/189; 81.0%), followed by chewing gum/lozenges (86/189; 45.5%) and saliva substitutes (82/189; 43.4%). Home remedies (e.g., oil or tea) were used by 62/189 (32.8%), saliva-stimulating mouth rinses by 44/189 (23.3%), and pharmacological sialogogues by 29/189 (15.3%); 17/189 (9.0%) reported using no measures.

**Fig. 4:**
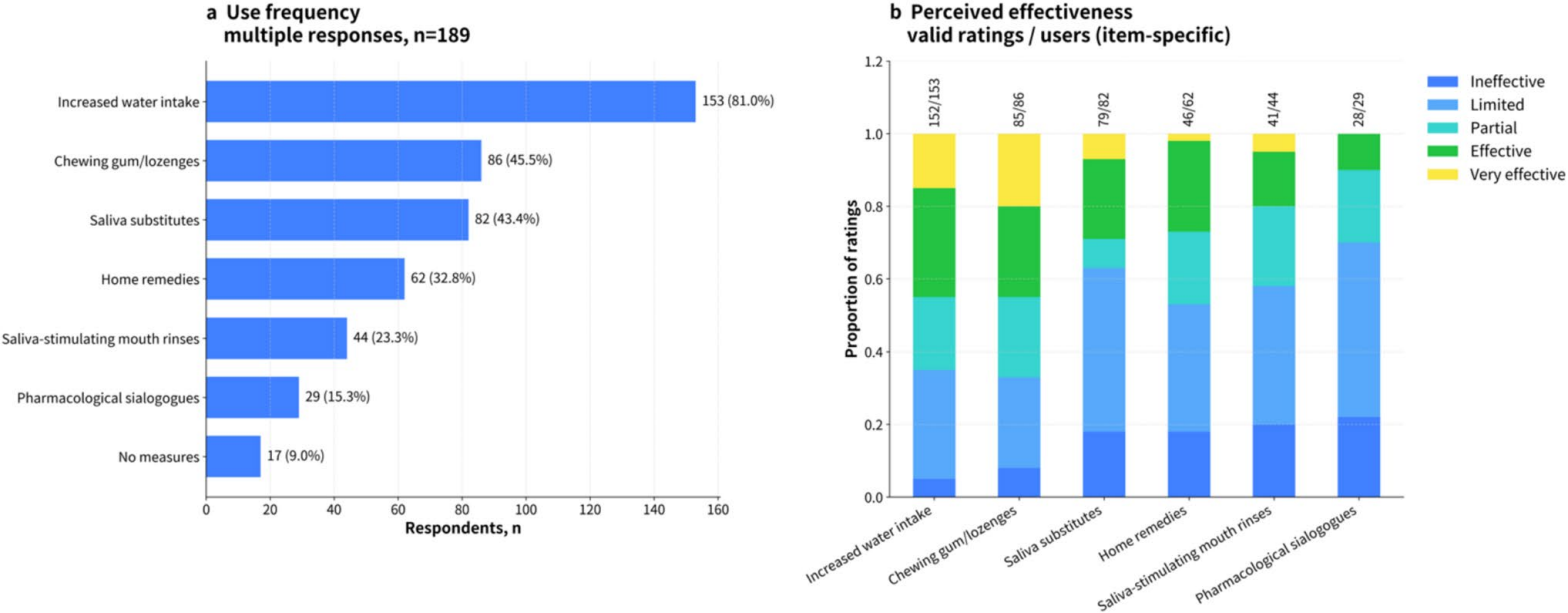
Xerostomia management and perceived effectiveness among xerostomia-affected respondents a Use frequency of xerostomia relief measures (multiple responses permitted, *n* = 189) Values indicate n (% of 189) b Perceived effectiveness ratings for each measure among users Bars show the proportion of ratings across a five-level scale (ineffective, limited, partial, effective, very effective) Numbers above bars indicate valid effectiveness ratings divided by the number of users endorsing the measure (valid/used), reflecting missing or “not applicable” effectiveness responses excluded from the denominator

Perceived effectiveness varied by measure and was analysed using item-specific denominators among users, excluding missing and “not applicable” effectiveness responses. Accordingly, effectiveness ratings were available for 152/153 water-intake users, 85/86 chewing gum/lozenge users, 79/82 saliva substitute users, 46/62 home remedy users, 41/44 saliva-stimulating mouth rinse users, and 28/29 pharmacological sialogogue users. The use frequencies and perceived effectiveness distributions are summarised in Fig. 4.

Behavioural strategies (*n *= 188; multiple responses permitted) most commonly included avoiding acidic/spicy/high-salt/high-sugar foods (116/188; 61.7%) and non-smoking or reduced tobacco use (116/188; 61.7%). Avoidance of alcohol and caffeinated/carbonated beverages was reported by 95/188 (50.5%). Twenty respondents (20/188; 10.6%) reported not wearing dentures at night, while 32/188 (17.0%) reported no avoidance behaviour. These behavioural strategies were self-reported in the study-specific questionnaire in relation to radiotherapy-associated xerostomia.

**Fig. 5:**
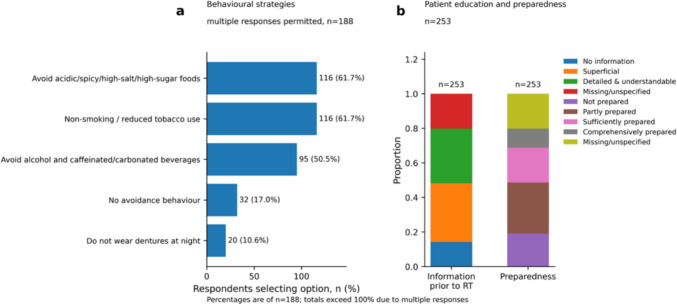
Behavioural strategies and patient education related to radiotherapy-associated xerostomia a Reported behavioural strategies to mitigate xerostomia symptoms (multiple responses permitted, *n* = 188) Values indicate the number and percentage of respondents selecting each option (n (%)); percentages refer to *n *= 188 and may exceed 100% across items due to multiple responses b Self-reported information on xerostomia prior to radiotherapy and perceived preparedness (*n *= 253) Stacked bars show the proportion of responses by category; “Missing/unspecified” denotes missing responses or responses not classifiable into the predefined categories

Regarding patient education and preparedness (*n *= 253), 86/253 (34.0%) reported having received only superficial information on xerostomia prior to radiotherapy, 80/253 (31.6%) reported detailed and understandable information, and 36/253 (14.2%) reported no information; the remaining 51/253 (20.2%) were missing/unspecified. With respect to preparedness (*n* = 253), 75/253 (29.6%) felt only partly prepared and 48/253 (19.0%) not prepared, while 51/253 (20.2%) felt sufficiently prepared and 28/253 (11.1%) comprehensively prepared; again, 51/253 (20.2%) were missing/unspecified. These items captured patient-reported counselling and information provided as part of radiotherapy care prior to treatment (e.g., by the radiotherapy team and/or written materials), rather than general knowledge. Exploratory Mann–Whitney U tests did not identify statistically significant sex-specific differences in perceived effectiveness for any relief measure (all *p* > 0.05; Fig. 5). In a post-hoc, exploratory comparison, self-reported xerostomia was more frequent among online- than clinic-recruited participants (93.9% vs. 71.8%; Fisher's exact *p* < 0.001). The cohorts also differed in tumour entity (oral cavity carcinoma 31.2% vs. 94.9%; *p* < 0.001), time since radiotherapy (> 2 years: 66.8% vs. 41.0%; *p* = 0.003), and age (≥ 70 years: 26.3% vs. 48.7%; *p* = 0.007), but not in sex (*p* = 0.49) or OHIP-G14 total score (24.8 ± 12.8 vs. 28.7 ± 14.3; *p* = 0.12). Within patients with oral cavity carcinoma, the higher prevalence in the online cohort persisted (100% vs. 70.3%; *p* < 0.001).

## Discussion

The present findings demonstrate a high prevalence of patient-reported radiation-induced xerostomia among head and neck cancer patients. With 90% of respondents reporting xerostomia in association with radiotherapy, the prevalence observed in this cohort lies at the upper end of rates reported in the literature. Comparable frequencies ranging from 64 to 93% have been described in clinical studies [21–23]. These findings confirm that xerostomia remains a common and clinically relevant late effect after curative radiotherapy for head and neck cancer, despite advances in treatment planning and delivery [24, 25].

While the observed prevalence is high, comparisons across studies should be interpreted cautiously, because definitions and assessment methods differ. By definition, xerostomia denotes the subjective sensation of oral dryness and can be ascertained only through patient self-report, whereas objective techniques such as sialometry quantify salivary gland hypofunction (hyposalivation) rather than xerostomia itself. Because subjective xerostomia and objectively measured hyposalivation are only partially correlated, studies that define dry mouth on the basis of measured salivary flow are not directly comparable with the present study, which assessed patient-reported xerostomia. This conceptual and methodological difference may contribute to the comparatively higher prevalence observed here. A further consideration is that the survey was explicitly framed as a xerostomia-focused questionnaire and was disseminated in part through xerostomia- and head-and-neck-cancer-specific patient self-help organisations. This design is likely to have introduced a response bias, because individuals personally affected by, or particularly concerned about, dry mouth may have been more inclined to participate. As a substantial proportion of participants were recruited through channels that preferentially attract individuals with a higher symptom burden, the resulting selection bias favours more pronounced complaints. For these reasons, the observed prevalence of 90% should not be interpreted as a population-level estimate of post-radiotherapy xerostomia, but rather as the symptom burden within a self-selected, symptom-aware survivorship cohort, and it almost certainly overestimates the true prevalence in the general head and neck cancer survivor population. The cohort was intentionally heterogeneous with respect to tumour entity, treatment pathways, and time since radiotherapy. Accordingly, the findings should be interpreted as reflecting overall survivorship burden rather than entity-specific effects.

As detailed radiotherapy and dosimetric data were not available, dose–response relationships between salivary gland exposure and subjective xerostomia severity could not be evaluated. Future studies should prospectively collect standardized radiotherapy and dosimetric parameters — in particular mean parotid and submandibular gland doses — and correlate these metrics with patient-reported xerostomia severity and oral health-related quality of life, in order to clarify dose–response relationships and to inform risk-adapted patient counselling [26].

Notably, xerostomia was frequently reported even years after completion of radiotherapy. In this cohort, 62.4% of respondents had completed radiotherapy more than two years before the survey, yet symptoms persisted in a large proportion, underlining the relevance of xerostomia as a long-term survivorship issue [12].

Among xerostomia-affected respondents, 35.9% reported maximal dryness at night and more than half reported sleep-related impairment. These findings suggest that xerostomia affects not only oral comfort but also nocturnal recovery and daily functioning. Sleep-related findings should be interpreted cautiously, as the questionnaire captured self-reported symptom presence rather than graded severity of sleep impairment [27, 28].

In addition to its high frequency, xerostomia was associated with substantial everyday burden. More than half of valid single-response cases (53.1%) rated the everyday burden of xerostomia as strong or very strong. This reinforces that xerostomia is not only frequent but also clinically consequential and likely to shape follow-up needs.

A key finding of this study is the significant association between xerostomia and impaired oral health-related quality of life (OHRQoL) as measured by OHIP-G14. Respondents reporting xerostomia had substantially higher OHIP-G14 total scores (mean 26.7) than those without xerostomia (mean 17.3), and this difference was highly significant (*p *< 0.001). Differences of this magnitude are clinically meaningful; methodological work suggests that changes of approximately 3 points on OHIP-14 may already represent clinically relevant change [29].

The multivariable linear regression model further identified xerostomia as an independent predictor of worse OHRQoL after adjustment for sociodemographic and clinical covariates. This is consistent with prior studies demonstrating that more severe radiation-induced xerostomia is associated with poorer patient-reported outcomes and diminished quality of life [22, 30, 31]. These results support the concept that radiogenic xerostomia has a distinct clinical burden and should not be regarded solely as a transient side effect but rather as a persistent survivorship condition with functional and sensory components.

Domain-level analyses showed that oral function and orofacial pain were the most affected OHRQoL domains. This is clinically plausible given the impact of xerostomia and post-radiotherapy changes on eating, speaking, and swallowing [32]. Functional impairment may also extend to taste-related limitations [33–35]. Aesthetic and psychosocial domains were comparatively less affected.

An additional noteworthy finding is the association between time since radiotherapy completion and OHRQoL in multivariable analysis, with higher OHIP scores reported at earlier post-treatment intervals. Longitudinal work suggests that both partial biological recovery and psychosocial adaptation processes (including “response shift”) may contribute to perceived improvements over time [36, 37]. Overall, the findings support a model in which post-radiotherapy OHRQoL is shaped by interacting factors, including salivary gland hypofunction, sensory alterations, and psychological adaptation. In combination, these factors determine how survivors perceive and manage long-term oral health consequences [38].

In this survey, most respondents reported using multiple strategies to alleviate xerostomia. Low-threshold, non-pharmacological approaches were most frequently used, particularly increased water intake, followed by chewing gum/lozenges and saliva substitutes. This pattern aligns with reports suggesting that patients tend to prefer practical, self-directed measures that can be readily implemented in daily life [39, 40]. Limited uptake of pharmacological sialogogues may reflect access issues, side effects, contraindications, or scepticism regarding benefit; however, causal explanations cannot be derived from the present dataset [38].

Perceived effectiveness varied substantially across measures, indicating heterogeneous symptom relief and the absence of a uniformly effective strategy. Because intervention use was self-selected and assessed cross-sectionally, these findings should be interpreted descriptively.

This study has several strengths that enhance its clinical relevance. Most importantly, it adopts a patient-centred perspective and combines a validated instrument (OHIP-G14) with a study-specific xerostomia questionnaire, enabling a broader assessment of xerostomia-related impact in daily life (e.g., eating, sleep, and coping strategies) beyond generic OHRQoL domains. At the same time, the non-standardized questionnaire component has not been externally validated, which may affect the reliability of individual items and limits direct comparability with other cohorts. However, the module was developed in collaboration with patient advocacy groups, supporting its patient-centred relevance and content coverage.

A further strength is the inclusion of respondents recruited through two complementary pathways (clinical tumour follow-up clinic and online dissemination), which likely captured a broader range of post-treatment experiences. However, this recruitment strategy also introduces limitations. As reported in the results, a post-hoc, exploratory comparison of the two cohorts revealed that online-recruited participants reported xerostomia markedly more often than clinic-recruited participants. This difference did not appear to be driven by tumour localisation, as it persisted when the analysis was restricted to patients with oral cavity carcinoma, and it is therefore more consistent with self-selection of symptom-aware individuals through the online, xerostomia-focused recruitment channels than with confounding by tumour site. As the clinic subgroup was small and the recruitment source was reconstructed post-hoc, this analysis is exploratory and hypothesis-generating; nonetheless, it indicates that online recruitment preferentially attracted individuals with a higher symptom burden, thereby inflating prevalence and severity estimates. Additionally, all clinical variables were self-reported and were not verified against medical records, which may have introduced recall bias and misclassification (e.g., tumour entity or stage).

Methodologically, the cross-sectional design precludes causal inference and does not allow evaluation of symptom trajectories or recovery over time. Although OHRQoL appeared to improve with increasing time since radiotherapy completion, longitudinal studies would be needed to distinguish biological recovery from psychosocial adaptation.

Another limitation is that intervention categories were assessed without product-level specification, preventing comparisons of specific agents or formulations. A formal response rate could not be calculated because the total number of individuals reached through the online dissemination pathways was unknown. Finally, item non-response and partial questionnaires resulted in varying denominators across analyses, and one questionnaire configuration error necessitated exclusion of multiple-response cases for a specific burden item, reducing analyzable sample size for that endpoint.

Despite these limitations, the study provides clinically meaningful insights into the real-world burden of radiation-induced xerostomia and its association with OHRQoL. The findings may help inform patient counselling prior to (chemo)radiotherapy by highlighting the potential for persistent long-term symptoms, setting realistic expectations, and discussing available coping and relief measures and their perceived effectiveness, thereby improving patient preparedness. Future research should employ prospective, longitudinal designs, larger multicentre cohorts, validated xerostomia-specific patient-reported outcome instruments, and—ideally—objective salivary function measures to strengthen causal interpretation and improve intervention evaluation.

## Conclusion

Radiotherapy-associated xerostomia is common in head and neck cancer patients and may persist long after treatment. In this cross-sectional survey, xerostomia was independently associated with poorer oral health-related quality of life, particularly affecting oral function and pain-related domains, and was frequently accompanied by impaired quality of sleep. Most patients relied on low-threshold behavioural coping strategies, whereas the perceived effectiveness of additional measures was heterogeneous. Beyond supporting routine patient-reported xerostomia monitoring and structured multidisciplinary supportive care during follow-up, these findings emphasize two forward priorities: further refinement of radiotherapy techniques and planning to maximize sparing of salivary glands and other relevant structures, and the development and rigorous evaluation of more effective interventions and products for xerostomia relief.

## Supplementary information

Below is the link to the electronic supplementary material.

ESM 1 (DOCX 25.2 MB)

## Data Availability

The datasets generated and/or analysed during the current study are available from the corresponding author on reasonable request.
