# Supplementary material for: Post-radiotherapy xerostomia and quality of life in head and neck cancer patients
Source: Support Care Cancer. 2026 Jun 27;34(7):698. doi: 10.1007/s00520-026-10948-9 (PMC13309429; doi:10.1007/s00520-026-10948-9)
Supplement: Supplementary file 1 — (DOCX 25.2 MB) [file 520_2026_10948_MOESM1_ESM.docx]

# Supplementary Appendix S1

German study questionnaire (original version) The questionnaire was administered in German. The complete original wording and response options are provided here to ensure transparency and reproducibility.

**Supplementary Table S1. Study-specific xerostomia questionnaire items (German original wording and English summary)**

| **Domain** | **Item (German original wording)** | **Item (English translation)** | **Response format** | **Response options (English)** | **Notes for analysis** |
| --- | --- | --- | --- | --- | --- |
| Xerostomia occurrence | Haben Sie nach der Strahlentherapie Erfahrungen mit Mundtrockenheit gemacht? | Have you experienced dry mouth after radiotherapy? | Single choice | Yes / No | Screening item |
| Onset timing | Wenn ja, wann trat die Mundtrockenheit erstmals auf? | If yes, when did dry mouth occur for the first time? | Single choice | During radiotherapy / Immediately after completion / A few weeks after completion / >6 months after completion / Not assessable due to other side effects | Asked only if xerostomia = Yes |
| Everyday burden | Wie stark belastet Sie die Mundtrockenheit im Alltag? | How strongly does dry mouth burden you in everyday life? | Ordinal (single choice intended) | Not at all / A little / Moderately / Strongly / Very strongly | Survey issue: multi-select was unintentionally enabled; item-specific analysis restricted to valid single-response cases |
| Time-of-day peak severity | Zu welcher Tageszeit empfinden Sie die Mundtrockenheit am stärksten? | At what time of day is dry mouth most severe? | Single choice | In the morning / During the day / In the evening / At night / All day / No specific time of day | Asked only if xerostomia = Yes |
| Functional impact (multiple response) | Haben Sie durch die Mundtrockenheit funktionelle Probleme? | Do you experience functional problems due to dry mouth? | Multiple response + optional free text | Speaking / Swallowing / Chewing / Tasting / Sleeping / Maintaining dental and oral health / Drinking liquids / No problems / Other (free text) | Multiple responses permitted |
| Saliva changes | Haben Sie Veränderungen der Speichelproduktion / Speichelbeschaffenheit bemerkt? | Have you noticed changes in saliva production/quality? | Single choice + optional free text | Less saliva / Thicker or more viscous saliva / No changes / Other (free text) |  |
| Sleep-related impact | Beeinflusst die Mundtrockenheit Ihren Schlaf? | Does dry mouth affect your sleep? | Single choice | Night-time awakenings due to dryness / Difficulty falling asleep or staying asleep / No sleep impairment | Asked only if xerostomia = Yes |
| Relief measures (use; multiple response) | Haben Sie in der Vergangenheit Maßnahmen ergriffen, um die Mundtrockenheit zu lindern? | Have you taken measures to relieve dry mouth? | Multiple response | Increased water intake / Chewing gum or lozenges / Saliva substitutes (gels, sprays) / Home remedies (e.g., cooking oil, tea) / Saliva-stimulating mouth rinses / Medications (sialogogues) / No measures | Multiple responses permitted |
| Relief measures (effectiveness; item-specific) | Wie wirksam sind die aktuell verwendeten Linderungsmaßnahmen für Sie? | How effective are the measures you currently use? | Ordinal (single choice per measure) | Ineffective / Limited / Partial / Effective / Very effective / Not applicable | Item-specific denominators; exclude missing and “not applicable” as predefined |
| Behavioural strategies (multiple response) | Haben Sie bestimmte Dinge vermieden, um die Mundtrockenheit zu lindern? | Have you avoided certain things to relieve dry mouth? | Multiple response | Avoid alcohol, caffeinated and carbonated beverages / Avoid acidic, spicy, very salty or sugary foods / Non-smoking or reduced tobacco use / Do not wear dentures at night / No avoidance measures | Multiple responses permitted |
| Patient information | Wie wurden Sie vor Beginn der Strahlentherapie über das Risiko von Mundtrockenheit und den Umgang damit aufgeklärt? | How well were you informed about dry mouth and coping strategies prior to radiotherapy? | Single choice | Detailed and understandable / Superficial / No information / Missing/unspecified | Percentages may be reported including missing/unspecified (see Results/Fig. 5) |
| Preparedness | Wie vorbereitet fühlten Sie sich auf das Auftreten von Mundtrockenheit und den Umgang damit? | How prepared did you feel for possible dry mouth and coping with it? | Single choice | Not prepared / Partly prepared / Sufficiently prepared / Comprehensively prepared / Missing/unspecified | Percentages may be reported including missing/unspecified (see Results/Fig. 5) |

*Note: For items allowing multiple responses, percentages reflect the proportion selecting each option and may exceed 100% across items.*
